# Brazilian biotechnology network - the challenge to innovate in biotechnology in Brazil

**DOI:** 10.1186/1753-6561-8-S4-O40

**Published:** 2014-10-01

**Authors:** Luiz Antonio Barreto de Castro

**Affiliations:** 1 The Brazilian Society for Biotechnology, Brasília, Brazil

## 

One of the most important roles the Brazilian Society of Biotechnology - SBBIOTEC can play is to organize the Biotechnology Sector. The Brazilian Biotechnology Network aims at the academic sector. The academic sector is represented by 48 Graduate Courses registered at CAPES (Coordenação de Aperfeiçoamento de Pessoal de Nivel Superior) /Ministry of Education, specifically in the area of Biotechnology. There are an even larger number of Graduate Courses related to Biotechnology but registered at CAPES in other areas such Biochemistry, Genetics. Pharmacology among others. So the Biotechnology academic sector is not irrelevant. However less than 1% of the scientific output in Biotechnology in Brazil reaches the market. The main goal of the Brazilian Biotechnology Network is to increase the visibility of the science that is produced in the Biotechnology sector in Brazil and related areas of application to catalyse the transfer of innovative scientific results to the industry. The academic and the private biotechnology sector do not interact. For this reason the number of scientists in Brazil hired by the private sector is small although Brazil graduates more than 1000 PhDs /year This reality differs considerably from the context in the United States that for this reason and others control 70% of the Biotechnology business in the world. In 2011 [1] CEBRAP and BRBIOTEC mapped 237 Biotechnology companies in Brazil. Efforts of this nature happen spasmodically. How was the Biotechnology sector structured in Brazil in 2011? 85% of the Biotechnology enterprises in Brazil had less than fifty employees and 56% of the revenues of these companies were around 1 million US $/year. Eighty % of these companies are located in three states: São Paulo, Minas Gerais and Rio de Janeiro. Most companies do not know that a network that I established in the North East of Brazil - RENORBIO deposited over one hundred and fifty patent applications in the last seven years and established 10 startup companies. This network and two others I established recently, one in the Amazonian region (Bioamazonia) and the other in West, the Savannah region in Brazil have web system and their graduate courses operate as Networks. RBB is intended to cover the whole country using the same software with some adjustments. Probably a larger number of patent applications and startup companies can be made visible if we extend the same software to the whole academic sector in Brazil thru the RBB. The software offer in addition: students, professors, disciplines, scientific outputs, patents and startup companies. Even small companies might be stimulated to hire PhDs holding patent applications. The software not only cites patent applications but the development of process in the patent Agencies. The RBB will be funded by the FAP/DF ( Fundação de Amparo a Pesquisa do Distrito Federal), not only the software to be developed by Hiragy TI but the monitoring of the system at SBBIOTEC : http://www.sbbiotec.org.br.

During the last three years I have contributed to the Bioentrepreneur blog of Nature Biotechnology: http://blogs.nature.com/tradesecrets/author/lbarreto. In my last contribution published in September 26 I state that the efforts to develop biotechnology in Brazil now exceeds three decades. This analysis that deals with Opportunities and Limitations for Biotechnology Innovation in Brazil is part of an eBook published by Bentham Books [2] I have written this e-book now because Brazil has emerged financially and now has more opportunities in biotech than previously. The book is intended for those who want to know some of the history of biotech in Brazil, and to ponder the power of this technology and the opportunities we now have in our hands. Brazil may become a relevant actor in this area internationally, taking advantage of some circumstances here that are not available in other countries, particularly our biodiversity.

Since the seventies Brazil started training young scientists in plant genetic engineering and molecular biology, hoping to incorporate this nascent technology into EMBRAPA's (Brazilian Enterprise for Agricultural Research) plant breeding efforts. This work I started in the 80s took place at The National Center for Genetic Resources and Biotechnology (CENARGEN) and added to the efforts of a dozen excellent geneticists. The soybean revolution in Brazil was brought about by Romeu Kihl; aluminum-resistant corn developed for the acidic "cerrado" soil was created by Ricardo Magnavacca; and the foundations of maize breeding had been previously done by the late Ernesto Paterniani. Eleuzio Curvello did cotton, and Alcides Carvalho, coffee, for 52 years of his life. Dalmo Giacometti and Silvio Moreira, citrus. Marcilio Dias and Hiroshi Ikuta are the fathers of the vegetable genetics; Raul Moreira tackled banana; Ady Raul da Silva, wheat. Finally Frederico Menezes Veiga, sugarcane in the North of Rio de Janeiro. Through all this, plant breeding has continuously built cultivars to feed our seed industry. High-tech seeds and low-cost farming practices were the result of seed laws and plant breeder's law in Brazil. The end result is that Brazil can competitively produce nearly 200 million tons of grain because it is cheap for the farmer. Brazil tried to follow the growth of commercial biology. In the '90s, Brazil introduced protections for intellectual property around genetics and plant breeding rights as aid before, but that efforts were not sufficient, because Brazil suffered through rampant inflation for decades even as it invested in Biotechnology.

The e-Book describes adjustments that must be made to assure the success of our investments, particularly to the laws and regulatory framework. The book investigates the immense possibilities of agricultural biotech in Brazil, partly because the mechanisms of public private interactions are well designed and in operation. The public perception in Brazil and many other countries has turned against genetic engineering for political and ideological reasons: when the first engineered soybean resistant to glyphosate was released commercially a campaign against transgenic plants prevented the application of this technology in agriculture for almost a decade in Brazil. However, the world adopted recombinant DNA technology in the pharmaceutical area and most products utilized internationally by the public in this industry (including Brazil) are genetically engineered. There is a twisted public perception problem in the Ag Biotech that has to be faced globally, particularly in Europe. The only solution is to focus biotech on the issues surrounding poverty (discussed in one of the e-book's chapters).

In the pharmaceutical industry in Brazil the context is quite different. Brazil lags behind many developed and emerging countries, and does not have an equivalent to EMBRAPA in the public pharmaceutical area. The area is growing, however (in 2011, up 14%), because the market is a demanding one. We import active principles from other countries and commercialize foreign products for our market here. So we are growing without innovation. There are initiatives that pull large, nationally funded companies together as consortia, and which propose "biosimilars" for monoclonal antibodies with expiring patents, but there is no innovation as an initiative. Brazil has no contract manufacturing organization, and not one CMO or cGMP-facility. Because of this, Brazil cannot scale up its pharmaceutical products, nor can it properly conduct the clinical studies needed for registration at ANVISA (http://portal.anvisa.gov.br/wps/portal/anvisa-ingles). Brazil has never produced a block buster or registered a pharmaceutical product at the FDA. Large corporations in general do not invest in Brazil, claiming Brazil's patent laws are not adequate. And finally, Brazil has no risk capital funds (Burrill & Co is a solitary actor).

There is a movement in the right direction, however. The government is supplying loans for biotech projects, allowing repayment in 10 to 15 years, with subsidized interest and the absence of capital payment for 3 to 5 years. Also, the federal government and private sector are funding scholarships to train 100,000 students in the next five years at all levels from high school to PhD. This is great news, but there are many more problems that need to be resolved in the coming decade. In 2005 Brazil sanctioned an Innovation Law. We estimated that in ten years 10 billion US$ would flow to the Biotechnology sector. The decade is almost over and funds never came by.
